# 16S rDNA microbiome composition pattern analysis as a diagnostic biomarker for biliary tract cancer

**DOI:** 10.1186/s12957-020-1793-3

**Published:** 2020-01-24

**Authors:** Huisong Lee, Hyeon Kook Lee, Seog Ki Min, Won Hee Lee

**Affiliations:** 1 Department of Surgery, Ewha Womans University College of Medicine, 1071 Anyangcheon-ro, Yangcheon-gu, Seoul, 07985 South Korea; 2 MD Healthcare Inc., Seoul, South Korea

**Keywords:** Metagenome, Microbiota, Gallbladder neoplasms, Biomarkers, Extracellular vesicles

## Abstract

**Background:**

The aim of this study is to investigate the composition of microbiota in biliary tract cancer patients and healthy adults by metagenome analysis and evaluate its potential values as biomarkers for biliary tract cancer.

**Methods:**

Patients who were diagnosed with biliary tract cancer or benign inflammation were enrolled in this study. The control group consisted of healthy adults who presented with no history of significant medical issues. We isolated bacteria-derived extracellular vesicles in the plasma. The microbiome composition was investigated with 16S rDNA metagenome analysis. We evaluated each microbiome to ensure suitability for the biliary tract cancer prediction model.

**Results:**

A total of 155 patients were included in this study: 24 patients with diagnosed biliary tract cancers, 43 diagnosed with cholecystitis or cholangitis, and 88 healthy adults. The microbiome composition pattern of the biliary tract cancer differed from the microbiome composition pattern seen in healthy adult group in beta diversity analysis. The percent composition of microbiota was found to be different from the phylum to genus level. Differences in the composition of the *Bifidobacteriaceae* and *Pseudomonaceae* families and *Corynebacteriaceae Corynebacterium*, *Oxalobacteraceae Ralstonia* and *Comamonadaceae Comamonas* species may be used to develop predictive models for biliary tract cancer.

**Conclusion:**

Biliary tract cancer patients have altered microbiome composition, which represents a promising biomarker to differentiate malignant biliary tract disease from normal control group.

## Introduction

Malignancy of the biliary tract is uncommon. However, it has poor prognosis for long-term survival. The prognosis differs depending upon the location and extent of disease [1]. For the treatment of biliary tract cancer, radical resection is necessary to improve survival outcomes [2]. However, the actual pathogenesis is not well understood. The chronic inflammation and liver fluke or typhoid fever have been thought to be a major cause of malignancy [3–7]. Recent reports suggest that certain bacteria, such as helicobacter, are associated with the development of gallbladder cancer [8, 9]. Nevertheless, it is unclear how the biliary tract cancer is related to the normal flora of our body. The microenvironments from normal flora can influence each other and even contribute to biliary tract cancer. Recently, techniques for meta-genome analysis have been rapidly developed. We can now analyze the microbiome from normal flora, which is considered to be important risk factors for genetic alteration of human [4, 5, 10–12].

Previous studies were focused on parasite or viral infection for hepatobiliary malignancies. However, recent studies emphasize the fact that there exists another element of human development, arising from human-microbiome interaction. The microbiota constantly influences human cells and even provides opportunities to refine prenatal and postnatal growth [13]. The microbiome produces fluent of bacteria-derived extracellular vesicles (EV). Bacteria can communicate with each other and human cell by the EVs, and it can be detected in the blood, urine, bile, and stool [14–16]. However, there is much room for error in performing genome analysis, and the quality of sample is important [12]. The EVs can maintain its shape for a long time and preserve bacteria-derived genomes. We can effectively analyze the composition of microbiota by filtering EVs [17]. In an in vivo study, EVs of *P. panacis* could infiltrated the gut barrier and moved to the target organs. Moreover, gut microbes influence host metabolic homeostasis and contribute to the pathogenesis of type 2 diabetes, which is characterized by insulin resistance [16].

## Microbiome composition as a novel biomarker

Microbe-derived EVs might be causative factors of various diseases. Recently, it has been determined that the EVs can even penetrate the blood-brain barrier. And there are documented differences in microbiome composition between autism spectrum disorder patients and the control group [18]. Moreover, there are studies to substantiate that microbiota is associated with colorectal cancer [19]. However, microbiome from bacteria-derived EVs was not investigated for biliary tract malignancy. The aim of this study is to compare the differences of composition of microbiota by metagenome analysis from bacteria-derived EVs. We expect that the composition of individual microbiome might be a novel biomarker to predict biliary tract cancer.

## Methods

### Subjects and plasma sample preparation

#### Inclusion and exclusion criteria

The patients were enrolled from a single tertiary hospital. This study complied with the Declaration of Helsinki and was approved by the Institutional Review Board of Ewha Womans University Mokdong Hospital (2017-07-031). Written informed consent was obtained from all patients before surgery including genetic analysis. The control group consisted of normal healthy adults who agreed with informed consent during health checkup. The control subjects had no history of malignant disease, nor any clinical findings suggestive of gastrointestinal problems or neuropsychiatric disorders. The control subjects of this study had not taken antibiotics, probiotics, or prebiotics in the 3 months immediately antedating the sample collection.

Patients undergoing surgery for benign inflammation or malignant biliary tract disease were assessed for the study. Patients were included if they were 20 or more years of age and had no history of cognitive dysfunction to interfere with informed consent. If any patient was found to have a previous history of cancer or a Karnofsky performance scale of less than 70, that patient would be automatically excluded [20].

The patients were divided into three groups: biliary tract cancer, benign inflammation, and a control group. The patients who are diagnosed with cholecystitis or cholangitis, based upon documented evidence of pathology, were classified into the benign inflammation group.

#### Sample collection

A trained and well-qualified data manager reviewed the pathologic diagnosis, which was confirmed by hepatobiliary pathologist. The blood samples were obtained using standard protocols. Blood samples were collected from the median cubital vein into Vacutainer tubes that contained EDTA tubes (BD, Franklin Lakes, NJ, USA). Then, the sample was centrifuged at 1500*g* for 10 min. The plasma was isolated and immediately preserved in a freezer.

### Metagenome pattern analysis

#### EVs isolation and DNA extraction from human plasma samples

EVs in human plasma were isolated using the differential centrifugation method as described previously [21]. For the extraction of DNA in isolated EVs, 1 μg (based on the protein amount) of the EVs was boiled at 100 °C for 15 min, and then it was centrifuged at 10000*g* for 20 min. The quality and quantity of the DNA were measured using the QIAxpert (QIAGEN, Germany).

DNA was extracted from EVs in human plasma using a PowerSoil DNA Isolation kit (MOBIO, USA). Bacterial genomic DNA was amplified with 16S_V3_F (5′-TCGTCGGCAGCGTCAGATGTGTATAAGAGACAGCCTACGGGNGGCWGCAG-3′) and 16S_V4_R (5′-GTCTCGTGGGCTCGGAGATGTGTATAAGAGACAGGACTACHVGGGTATCTAATCC-3′) primers, which are specific for V3-V4 hypervariable regions of 16S rDNA gene [22]. The libraries were prepared using polymerase chain reaction (PCR) products according to MiSeq System guide (Illumina, USA) and quantified using a QIAxpert (QIAGEN, Germany). After PCR products were extracted and quantified, equimolar ratios from each mixture were pooled and sequenced on a MiSeq (Illumina, USA) according to the manufacturer’s recommendations.

#### Taxonomic assignment

Raw pyrosequencing reads obtained from the sequencer were filtered according to the barcode and primer sequences using MiSeq (Illumina, USA). Taxonomic assignment was performed by profiling program MDx-Pro ver.1 (MD Healthcare, Korea). To select 16S rDNAs, all the sequence reads were compared to the GREENGENES. Sequence reads that had a similar sequence with more than 100 bit score and less than 1.0 *E* value were admitted as partial 16S rDNA sequences. Taxonomy-based analyses were performed using GREENGENES database [23, 24].

#### Sample size estimation and statistical analysis

This is the first study for metagenome analysis to compare the differences of composition of microbiome between the microbiome of patients afflicted with diagnosed biliary tract cancer and those suffering from benign biliary tract disease. Therefore, we cannot estimate the exact sample size. A previous study, which investigated microbiome composition, enrolled 20 patients in the patient group [18]. The initial goal of this study was to enroll more than 20 patients with diagnosed biliary tract cancer, as well as in excess of 40 benign inflammation cases. The control group was matched to the biliary tract cancer group and benign inflammation group with regard to chronologic age and sex. We performed logistic regression analysis with a randomized sampling of 30% of patients from each group. In the univariate analysis, we selected the top 5 microbiomes which were statistically associated with biliary tract cancer. Also, we performed multivariate analysis and tried to derive a prediction model. And we tried to validate the model with validation set.

The categorical variables are presented as number (percentage) and compared with *χ*^2^ test. The continuous variables are presented as the mean ± SD and were compared using a Kruskal-Wallis test or ANOVA test. A *p* value < 0.05 was considered statistically significant.

#### Biliary tract cancer prediction model development

To reduce the selection bias, patients in the model development set were randomly allocated into “training” and “validation” sets. Four fifth of cases were assigned to the training set, and the other cases were assigned for test set [25].

We sorted the individual microbiome in the order of proportion and investigated the differences between the biliary tract cancer group and control group to find out potential markers with microbiome percent composition analysis. Significant microbiome was evaluated from phylum to family level. For the selection of the bio-markers, we considered relative abundances of operational taxonomic units (OTUs) at genus level. All prediction models include age and sex as covariates. First, we selected the candidate biomarkers with *p* value < 0.01, fold change > 2-fold, and the average of relative abundances as > 0.1%. Next, we used the Akaike Information Criteria (AIC) to infer a microbiome that is likely to be a biomarker candidate as a step-by-step selection method that compares predictive models with variable numbers of variables. Finally, the diagnostics model was calculated with the logistic regression. The regression coefficient (*b*) of the logistic regression model was regarded as the log odds ratio (OR).

$$ p=\frac{e^{\beta_0+{\beta}_1{x}_1+\cdots +{\beta}_7{x}_7}}{1+{e}^{\beta_0+{\beta}_1{x}_1+\cdots +{\beta}_7{x}_{7.}}} $$

We developed the biliary tract prediction model with the coefficients based on the training set. It was validated by receiver operating characteristic (ROC) curve and calculation of the area under the ROC curve (AUC) with the validation set.

## Results

### Patient demographics

An aggregate 25 samples of biliary tract cancer were investigated, and one case was excluded after quality control testing. And 45 samples of benign inflammation group were evaluated, and two cases were excluded due to contamination. For the control group, 88 normal healthy adults were matched to the benign inflammation and biliary tract cancer group. Within the biliary tract cancer group, there were seven cases of diagnosed gallbladder cancers, nine intrahepatic cholangiocarcinoma, and eight extrahepatic cholangiocarcinoma. The average age of the biliary tract group, benign inflammation group, and control group were 69.8 ± 10.7 years, 55.4 ± 15.5 years, and 54.4 ± 12.8 years old, respectively (Table 1).

**Table 1:** Characteristics of patients

Characteristics		Biliary tract cancer (*n* = 24) (%)	Benign inflammation (*n* = 43) (%)	Control (*n* = 88) (%)	*p* value^*^
Sex	Male	15 (63)	15 (35)	37 (42)	0.086
	Female	9 (37)	28 (65)	51 (58)	
Age (years) (mean ± SD)		69.8 ± 10.7	55.4 ± 15.5	54.4 ± 12.8	< 0.001

*SD* standard deviation

*Chi-square test or Kruskal-Wallis test

### Differences of microbiome composition in bacteria-derived EVs

**Fig. 1:**
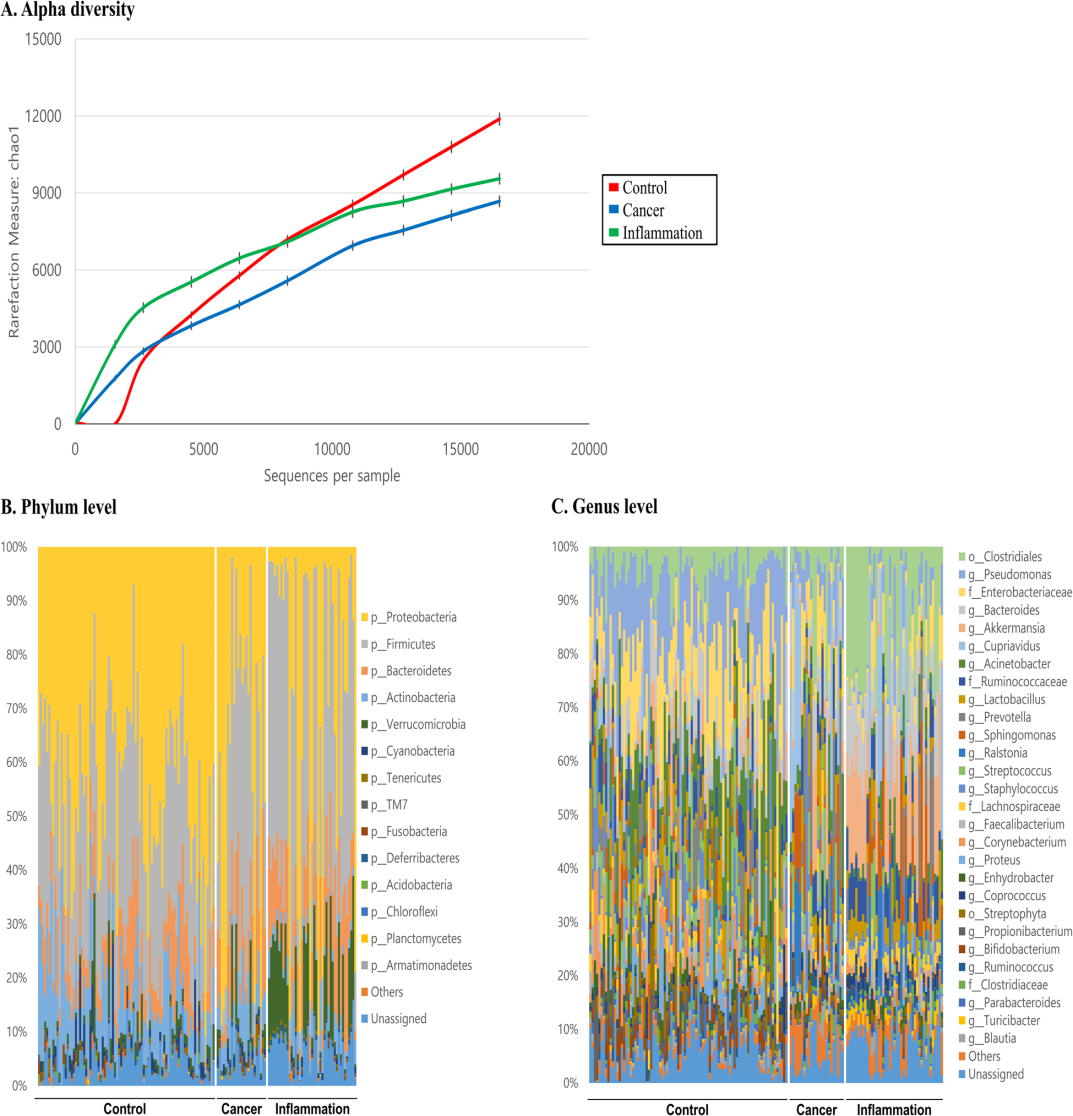
**a** The alpha diversity curves. Rarefication curves representing the mean operational taxonomic units over the identified sequences of variable regions of 16S rDNA gene in the biliary tract cancer, benign inflammation, and control group. **b** Microbiome composition analysis in phylum level. **c** Microbiome composition analysis in genus level

We isolated the bacteria-derived EVs. Then, variable regions of the 16S rRNA genes were amplified by PCR. We were able to identify over 7000 OTUs by subsequent DNA sequencing analysis in each biliary tract cancer patient, benign inflammation patient, and each individual in the control group. Among the identified OTUs, we assigned 41 OTUs at the phylum level, 102 OTUs at the class level, 203 OTUs at the order level, 384 OTUs at the family level, and 939 OTUs at the genus level. There were no differences in the alpha diversity. Therefore, we could perform further quantitative analysis and compare the microbiome composition (Fig. 1).

**Fig. 2:**
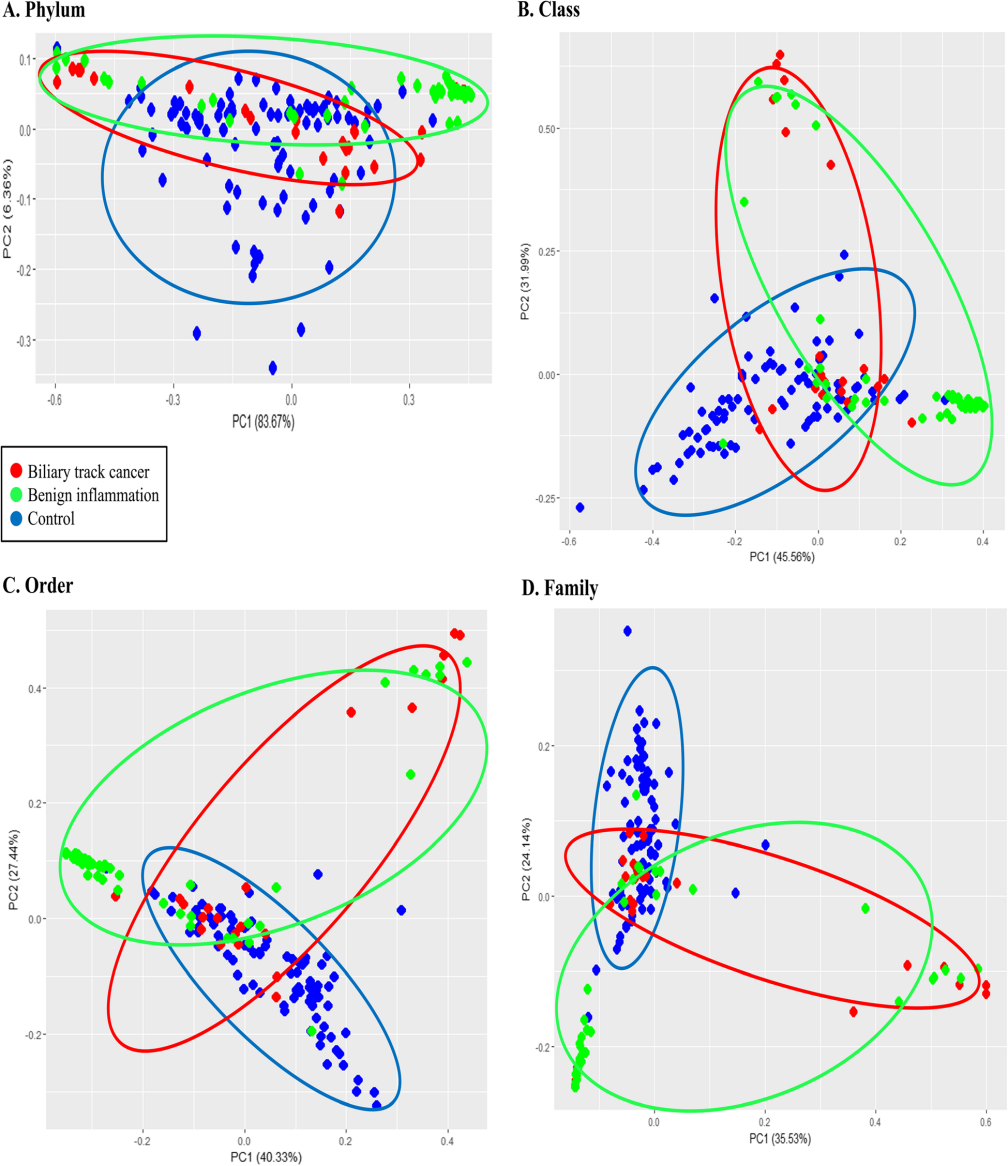
The beta diversity sheet of metagenome pattern from the phylum to family level. Principal component analysis of microbiota diversity based on the weighted UniFrac distance and Bray-Curtis dissimilarity. Biliary tract cancer (red), benign inflammation (green), and control (blue). **a** Phylum level, **b** Class level, **c** Order level, **d** Family level

According to the taxonomy-based analysis, there were differences in the microbiome composition in beta diversity. We performed the principal component analysis of microbiota diversity based on the weighted UniFrac distance and Bray-Curtis dissimilarity. According to the dot pattern, we were able to roughly categorize the groups. As a result of the dot pattern, the microbiome pattern of the biliary tract cancer group was different from that of the normal healthy group, but similar to that of the cholecystitis group (Fig. 2). We analyzed the percent composition of individual microbiome from phylum to family level. Sequence readings of EVs-based 16S rDNA indicated that the top five members of the phyla *p_Proteobacteria*, *p_Firmicutes*, *p_Actinobacteria*, *p_Bacteroidetes*, and *p_Cyanobacteria* comprised 94.7% of the identified OTUs in healthy subjects, whereas these members covered 93.8% of the total OTUs in the biliary tract cancer individuals. In the benign inflammation group, the proportion of top five phyla was 88.1% and lower than the other two groups. We could therefore surmise that the patients with cholecystitis or cholangitis have altered phyla composition. The occupancy of *p_Proteobacteria* of the biliary tract cancer patients, benign inflammation patients, and in the control group was 38.4%, 30.0%, and 43.9%, respectively. On the other hand, the proportion of *p_Firmicutes* was 33.1%, 39.1%, and 28.2%, respectively.

**Fig. 3:**
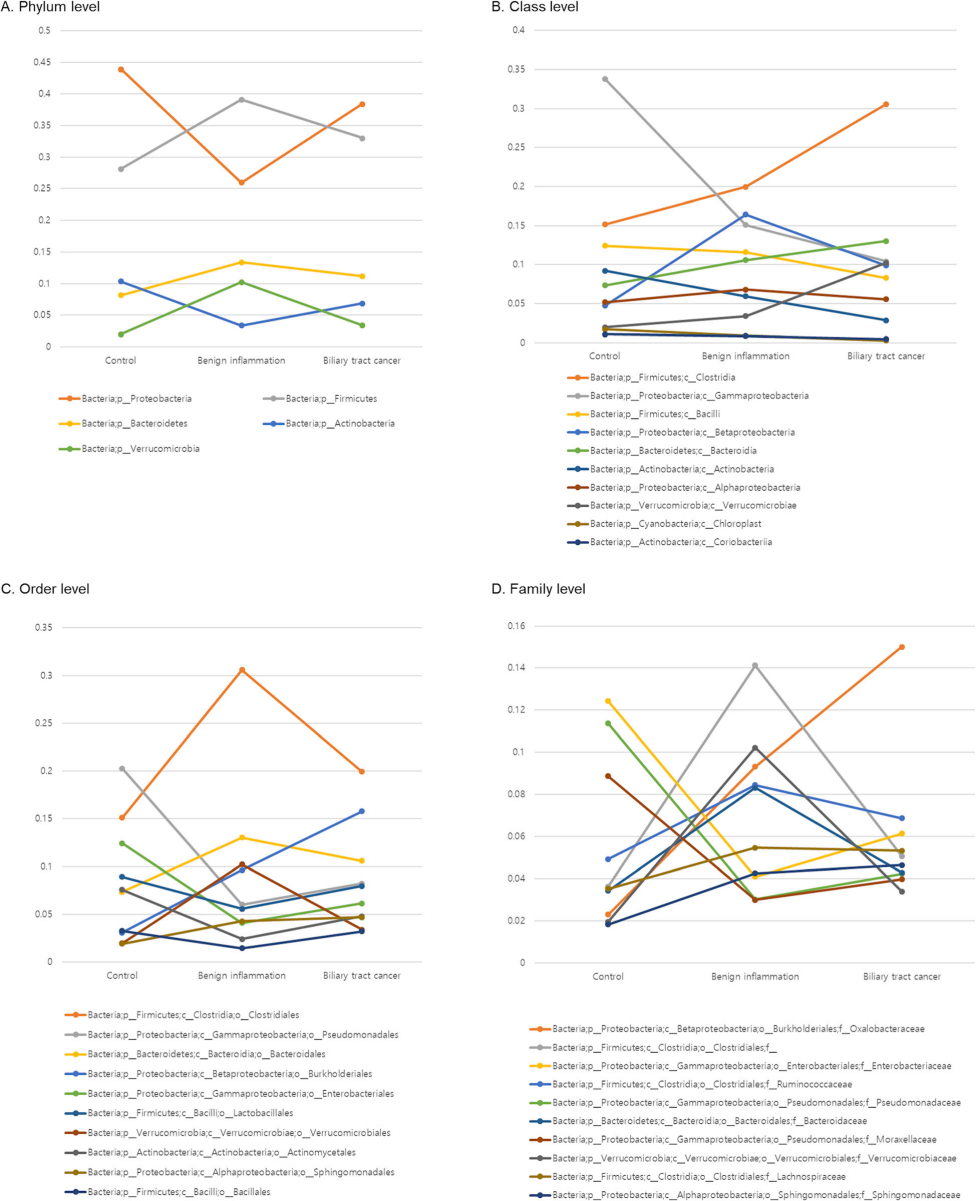
The percent composition of microbiota from phylum to family level. Overall composition of microbiota was compared. The top five subjects in phylum level and top ten subjects from class to family level are presented. **a** Phylum level, **b** Class level, **c** Order level, **d** Family level

In class level analysis, the proportion of *c_Clostridia* of biliary tract cancer, benign inflammation, and the control group was 30.1%, 19.9%, and 15.1%, respectively, although the proportion of *c_Gammaproteobacteria* was 10.4%, 15.1%, and 33.8%, respectively (Fig. 3).

### Biliary tract cancer prediction model

We could identify five important microbiomes that the occupancy rate was significantly increased or decreased on the percent composition analysis. The compositional differences of *Bifidobacteriaceae* family and *Oxalobacteraceae Ralstonia* was found to be a significant positive marker, and the *Pseudomonaceae* family, *Corynebacteriaceae Corynebacterium*, and *Comamonadaceae Comamonas* species were found to be significant negative markers to differentiate biliary tract cancer patients from the individuals in the control group. We developed the biliary tract cancer prediction model with these five variables in company with chronologic age and sex based on the training set (Table 2).

**Table 2:** The biliary tract cancer prediction model. The compositional differences of *Bifidobacteriaceae* and *Pseudomonaceae* families and *Corynebacteriaceae Corynebacterium*, *Oxalobacteraceae Ralstonia*, and *Comamonadaceae Comamonas* species were significant positive or negative markers to differentiate biliary tract cancer from control group

X	Variables	*B*
X1	Age	1.376
X2	Sex	− 12.98
X3	p_Actinobacteria; c_Actinobacteria; o_Bifidobacteriales; f_Bifidobacteriaceae	567,500
X4	p_Proteobacteria; c_Gammaproteobacteria; o_Pseudomonadales; f_Pseudomonadaceae	− 58,450
X5	p_Actinobacteria; c_Actinobacteria; o_Actinomycetales; f_Corynebacteriaceae; g_Corynebacterium	− 1943
X6	p_Proteobacteria; c_Betaproteobacteria; o_Burkholderiales; f_Oxalobacteraceae; g_Ralstonia	2881
X7	p_Proteobacteria; c_Betaproteobacteria; o_Burkholderiales; f_Comamonadaceae; g_Comamonas	− 39,210
	Intercept	− 18.35

**Fig. 4:**
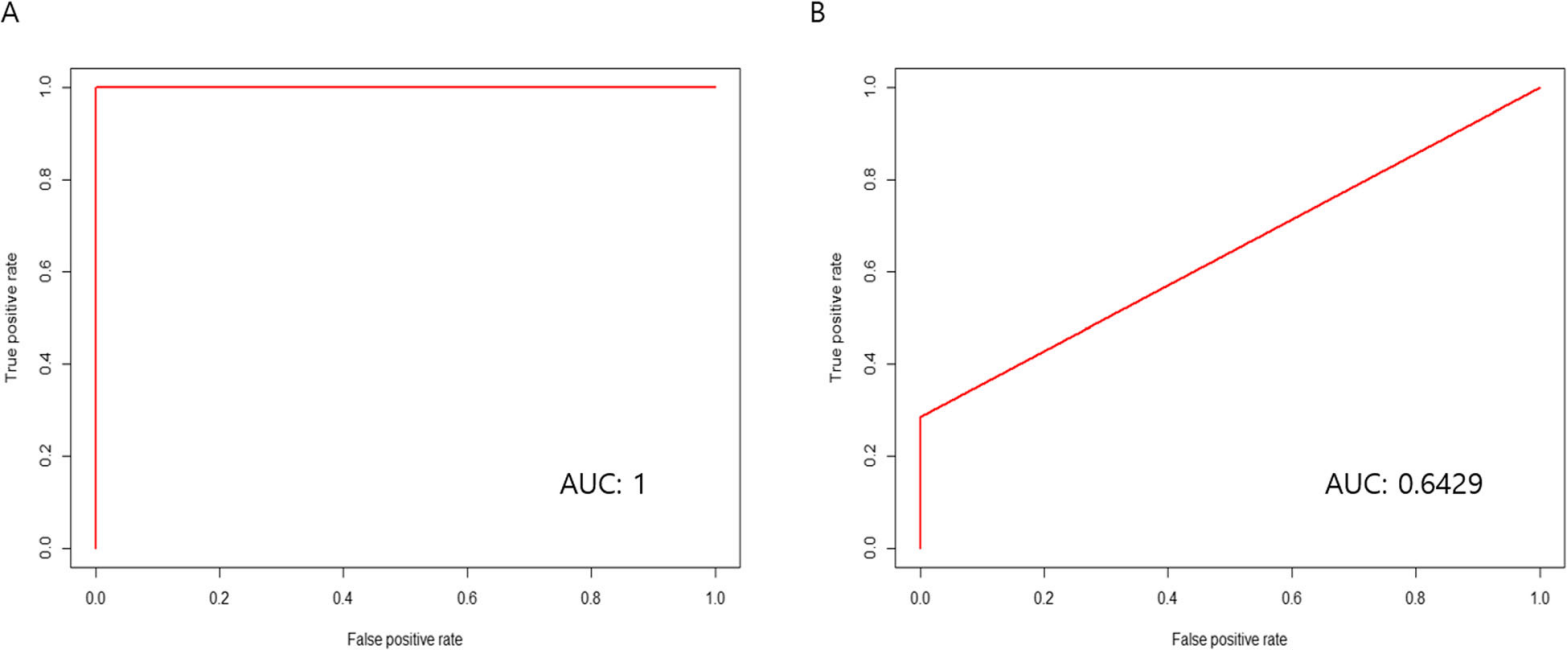
Receiver operating characteristic (ROC) curve analysis of the biliary tract cancer according to microbiome pattern. **a** Between normal healthy group and biliary tract cancer group. **b** Between cholecystitis group and biliary tract cancer group

We validated the prediction model with ROC curve, and the AUC was one. The composition of these five markers was obviously different upon comparison made between the biliary tract cancer patient and the control group. The accuracy was 1.0000 (confidence interval, 0.8518 to 1.0000), kappa value was 1.0000, and *p* value was 0.0035. The sensitivity of the model was 1.0000, and the specificity was 1.0000. The positive prediction value was 1.0000, and the negative prediction value was 1.0000. The balance accuracy was 1.0000 (Fig. 4a).

We compared the microbiome composition between the biliary tract cancer and cholecystitis groups. The composition of *Bacillus* and *Bifidobacterium* genus were different. In order level, *Anaeroplasmatales*, *Erysipelotrichales*, and *Bacteroidales* were different. However, there was no statistically significant difference in ROC curve analysis (Fig. 4b).

## Discussion

The microbiota is thought to be a component of the human body and a source of genetic diversity and modifier of disease [11, 26]. Moreover, recent studies investigated and revealed that there is extremely active interaction and synergistic effect between the microbiota and human host cells by EVs. This mechanism has been recently spotlighted as a pathogenetic mechanism of various diseases [14, 15, 27–29].

To the best of our knowledge, this study is the first attempt to analyze the composition of microbiome from EVs in patients with biliary tract cancer. Ultimately, we were able to successfully analyze the composition of microbiome in biliary tract cancer and benign inflammation patients compared with normal healthy adult group. We determined that specific families or species were extremely increased or decreased in the biliary tract cancer group, when compared to the control group. The compositional differences of *Bifidobacteriaceae* and *Pseudomonaceae* families and *Corynebacteriaceae Corynebacterium*, *Oxalobacteraceae Ralstonia*, and *Comamonadaceae Comamonas* species were found to be significant markers to make a biliary tract prediction model. In this study, however, the microbiome composition was similar between patients with cholecystitis and biliary tract cancer. Chronic inflammation such as chronic cholecystitis or cholangitis is also associated with cancer development. Previous studies have reported that *Helicobacter* species are associated with the development of both gallstones and gallbladder cancer [9, 30, 31]. Based on these results, it will be possible to use the microbiome pattern as a marker of cancer diagnosis in the future.

Traditionally, chronic cholecystitis or cholangitis is thought to be associated with malignant transformation [32]. In previous studies, certain bacteria were reported to be associated with the development of gallstone and biliary tract cancer [8, 9, 30, 31]. However, the actual mechanism of transformation has not yet been identified. We believe that the metagenome analysis helps us to explain the actual pathogenesis by which inflammatory changes transform to progressive malignancy. The metagenome analysis from blood sample represents the altered microbiota composition. Previous studies have demonstrated, from the blood and urine, EVs which were partially consistent with the results from fecal samples [18, 33]. We can explore and investigate the alteration of gut microbiota by analyzing the metagenome analysis from the blood or urine samples, indirectly. The composition alteration may have role in the manifestation of various types of pathology. Microbiome is greatly affected by dietary habits, BMI, and blood lipid level [34]. However, the variables were not analyzed nor matched in this study. Therefore, further study is necessary to overcome the limitations of this study.

There are many published studies addressing the issue of genetic mutations associated with biliary tract cancer. Numerous kinds of genetic mutations had been investigated, and the KRAS, BRAF, TP53, SMAD, and p 16 (INK4) mutations are well known factors for cholangiocarcinoma pathogenesis. And the inflammatory cytokines such as interleukin-6, transforming growth factor-beta, tumor necrosis factor-alpha, and platelet-derived growth factors are also an important factors of cancer pathogenesis [35–38]. Although we were unable to identify the precise genetic mutations associated with the composition of microbiome, the subject was, and remains, very much worthy of the effort because the microbiome is now thought to be the major cause of genetic diversity. As more and more pathophysiological roles for EVs are recognized, it is considered for potential novel targets for treatment. Moreover, modified and engineered extracellular vesicles are likely to have applications in macromolecular drug delivery [15, 17, 39].

Although the results of this study confirm the encouraging results, further studies are needed. In this study, random sampling was performed to construct a test set and a validation set. However, since only internal validation has been performed, external validation is needed in the future. The results of the study showed that the AUC converged to 1, which may be a statistically over-fitting error. In this study, the number of cases was relatively small. More cases will need to be analyzed in order to find out the proper bacteria that have diagnostic value among numerous strains. Nevertheless, this study is of great significance in finding hopeful clues about the diagnostic value of microbiomes in the future.

## Conclusion

The microbiome composition of the biliary tract cancer patients and normal healthy adults is found to be different when compared. We were able to develop a biliary tract cancer prediction model from the compositional differences of *Bifidobacteriaceae and Pseudomonaceae* families and *Corynebacteriaceae Corynebacterium*, *Oxalobacteraceae Ralstonia*, and *Comamonadaceae Comamonas.* Biliary tract cancer patients seem to have altered gut microbiota, which is promising biomarker to differentiate malignancy from the physiology of the normal control group. However, there was no significant difference in microbiome composition between the cholecystitis patients and gallbladder cancer patients. Therefore, further study is necessary to confirm the differences of microbiome composition between biliary tract cancer and benign inflammation. Moreover, the genetic mutation of cancer cells warrant investigation, to confirm the cause and effect.

## Data Availability

The datasets generated and/or analyzed during the current study is available from the corresponding author on reasonable request.

## Abbreviations

AIC: Akaike information criterion
AUC: Area under the ROC
EV: Extracellular vesicle
OR: Odds ratio
OTU: Operational taxonomic units
PCR: Polymerase chain reaction
ROC: Receiver operating characteristic
